# Asian race and origin have no clinically meaningful effects on polatuzumab vedotin pharmacokinetics in patients with relapsed/refractory B-cell non-Hodgkin lymphoma

**DOI:** 10.1007/s00280-020-04119-8

**Published:** 2020-08-08

**Authors:** Rong Shi, Tong Lu, Grace Ku, Hao Ding, Tomohisa Saito, Leonid Gibiansky, Priya Agarwal, Xiaobin Li, Jin Yan Jin, Sandhya Girish, Dale Miles, Chunze Li, Dan Lu

**Affiliations:** 1 Genentech Inc., South San Francisco, CA USA; 2 Chugai Pharmaceutical Co., Ltd., Tokyo, Japan; 3 QuantPharm LLC, North Potomac, MD USA

**Keywords:** Ethnic sensitivity assessment, NHL, Non-hodgkin lymphoma, PK, Polatuzumab vedotin

## Abstract

**Purpose:**

The CD79b-targeted antibody–drug conjugate polatuzumab vedotin (pola), alone and with chemoimmunotherapy, has clinical efficacy and a tolerable safety profile in B-cell non-Hodgkin lymphoma (B-NHL). We assessed (a) whether exposure from global studies of pola is comparable to Asian patients, and (b) if the recommended pola dose is appropriate in Asian patients based on exposure.

**Methods:**

The pharmacokinetics (PK) of pola in Asian and global populations was characterized for three analytes (antibody-conjugated monomethyl auristatin E (MMAE) [acMMAE], total antibody, and unconjugated MMAE) in five phase 1b/2 single-agent and combination studies in B-NHL patients (JO29138 [JAPICCTI‐142580], DCS4968g [NCT01290549], GO27834 [NCT01691898], GO29044 [NCT01992653], and GO29365 [NCT02257567]). PK data were compared between Japanese phase 1 JO29138 (JAPICCTI‐142580) and global phase 1 DCS4968g (NCT01290549) studies and between Asian and non-Asian patients in the randomized relapsed/refractory B-NHL cohorts of the phase 1b/2 study GO29365 (NCT02257567). A population PK (popPK) model was used to assess the effects of Asian race and region on acMMAE and unconjugated MMAE exposure.

**Results:**

PK non-compartmental analysis (NCA) parameters for the key analyte acMMAE in the Japanese JO29138 (JAPICCTI‐142580) and global phase 1 DCS4968g (NCT01290549) studies were similar. In GO29365 (NCT02257567), the phase 1b/2 combination study, mean exposure to the analytes was generally lower in Asian patients (by ~ 9.9 to 17.5%), but not to a clinically meaningful extent. Overall, the popPK model further suggested comparable PK in Asian patients with B-NHL (race or region) versus non-Asian patients.

**Conclusion:**

Race has no clinically meaningful effect on pola PK. These results (and observations from efficacy/safety exposure–response analyses) support no pola dose adjustments are warranted for Asian patients with DLBCL.

**Electronic supplementary material:**

The online version of this article (10.1007/s00280-020-04119-8) contains supplementary material, which is available to authorized users.

## Introduction

The antibody–drug conjugate (ADC) polatuzumab vedotin (pola; Polivy^®^) was approved by the United States Food and Drug Administration in June 2019 for use in relapsed/refractory (R/R) diffuse large B-cell lymphoma (DLBCL), in combination with bendamustine and rituximab (BR), for patients who have received two or more prior therapies [1]. DLBCL is the most common subtype of B-cell non-Hodgkin lymphoma (B-NHL) and, although often curable, approximately 40% of patients are refractory to, or will relapse after, standard front-line chemoimmunotherapy with rituximab plus cyclophosphamide, doxorubicin, vincristine, and prednisone (R-CHOP) [2, 3]. For these patients, the standard of care remains autologous stem cell transplantation (ASCT) which also offers a curative approach. However, many DLBCL patients are ineligible for such therapy due to age, performance status, or comorbidities [4]. Unfortunately, the prognosis remains poor for patients with DLBCL who are not transplant eligible, do not respond to salvage therapy, or relapse after ASCT with a median overall survival (OS) of approximately 6 months [5, 6].

For many years, progress in identifying more effective therapies for patients with R/R DLBCL has been very limited. More recently, anti-CD19 chimeric antigen receptor T-cell therapies have become available but are associated with significant toxicity [7] and are only available at selected medical centers. As such, pola may help to address a long-standing unmet treatment need in these patients. Pola is an ADC comprising the potent cytotoxic microtubule inhibitor monomethyl auristatin E (MMAE) conjugated to an anti-CD79b monoclonal antibody via a protease-cleavable linker. MMAE is preferentially delivered to B cells expressing CD79b, which results in anti-cancer activity against B-cell malignancies [8]. Pola (1.8 mg/kg by an intravenous [IV] infusion every 3 weeks [Q3W]) was approved based on data from a randomized cohort of a phase 1b/2 study (GO29365 [NCT02257567]), in which pola in combination with BR (pola-BR) improved the outcomes of patients with R/R DLBCL compared with BR, and had a tolerable safety profile [9]. The study met its primary endpoint, with pola-BR inducing higher complete response rates at end of treatment compared with BR (40.0% vs. 17.5%; *p* = 0.026). Median progression-free survival (PFS) was significantly longer with pola-BR than with BR (9.5 vs. 3.7 months; stratified hazard ratio [HR] 0.36 [95% confidence interval (CI) 0.21–0.63]; *p* < 0.001); median OS was also improved (12.4 vs. 4.7 months; HR 0.42 [95% CI 0.24–0.75]; *p* = 0.002). Pola is currently being investigated in the front-line DLBCL setting in combination with rituximab, cyclophosphamide, doxorubicin, and prednisone (R-CHP; with the cytotoxic component substituting for vincristine in the R-CHOP regimen [comparator arm]) in the ongoing phase 3 multicenter, randomized, double-blind placebo-controlled trial, POLARIX (NCT03274492) [10].

Ethnic diversity in drug efficacy and safety can lead to the need to adjust the recommended dose across different populations. Such ethnic differences are more applicable to small molecules rather than large molecules such as monoclonal antibodies (mAbs). For example, the approved starting dose for single-agent docetaxel is lower in Asian than in Western countries due to the higher rates of hematologic toxicity reported in Asian patients [11]. Similarly, for doxorubicin, higher hematologic toxicity was found in Chinese patients; Fan and colleagues [12] reported on a CBR3 variant where homozygous patients had a lower area under the concentration–time curve (AUC) ratio of a metabolite of doxorubicin, with these patients showing more hematologic toxicity but better tumor response [12]. This variant is found more commonly among Chinese patients than in Indian and Caucasian populations. A comprehensive review of the pharmacokinetics (PK) of approved therapeutic mAbs in Japan concluded that PK of mAbs is largely driven by body weight and antigen level, which supports body weight-based dosing of an ADC when body weight contributes significantly to the inter-subject variability of clearance [13]. This is supported by other studies in which ethnicity did not affect responses to rituximab-containing regimens in B-NHL [14, 15].

ADCs contain an antibody, a linker, and a cytotoxic small molecule, and represent a special class of drugs when compared with the small or large molecules previously investigated in ethnic groups. Upon binding to the target CD79b, pola is internalized in the target cells, and unconjugated MMAE is then released by proteolytic cleavage of the linker [8, 16]. Unconjugated MMAE is expected to be mostly eliminated in feces via the biliary/fecal route, and urinary excretion is a minor elimination pathway. MMAE is mostly eliminated unchanged in feces and urine and is only partly metabolized [Genentech, Inc., data on file]. For the elimination portion that is metabolized, MMAE is mostly metabolized via cytochrome P450 3A4 (CYP3A4) [17]. Ethnic-related polymorphism in metabolism enzymes has been reported as a possible explanation for requiring different doses of small molecules in Asian patients; MMAE elimination will be evaluated in the current report in this context. Brentuximab vedotin, an ADC with the same linker and cytotoxic payload (MMAE) as pola, was evaluated in a population PK (popPK) model including 30 Asian patients (8% of a total of 380 patients with lymphomas), where body size, but not race, showed a strong effect on the clearance of MMAE; additionally, the conjugate drug clearance was not affected by race [18]. The exposures of pola related analytes (total antibody, acMMAE and unconjugated MMAE) increased proportionally over a pola dose range from 0.1 to 2.4 mg/kg [8]. The acMMAE terminal half-life is approximately 12 days (95% CI 8.1–19.5 days) at Cycle 6. The unconjugated MMAE terminal half-life is approximately 4 days after the first pola dose [1].

As pola has only recently been approved, there are limited data on how ethnic differences in patients with DLBCL may affect treatment outcomes with pola. PK, safety, and efficacy profiles in a global phase 1 dose-escalation study (DCS4968g; NCT01290549) in 34 patients with R/R B-NHL receiving pola alone or in combination with rituximab [8] have been compared with results from a phase 1 study conducted in seven Japanese patients with R/R B-NHL (JO29138; JAPICCTI‐142580), with preliminary results showing similar safety, PK, and efficacy [19].

In the US, pola is currently approved at the dose of 1.8 mg/kg Q3W, in combination with BR, for patients with R/R DLBCL who have received at least two prior therapies. The main objective of the current analysis was to conduct an ethnic sensitivity assessment of pola PK to systematically evaluate whether the PK of pola is similar in Asian and global populations, based on data from patients with B-NHL. The PK comparisons are discussed in the context of the exposure–response (efficacy/safety) relationship of pola in Asian and global populations. Here, we sought to determine whether any dose adjustment is required for Asian patients, both for the currently approved indication for pola in patients with R/R DLBCL and potentially for other B-NHL indications that may be approved in the future.

## Materials and methods

### Data sources and study populations

PK analyses were conducted on data from five clinical trials in which pola was administered either as a single agent or in combination with other therapies in patients with B-NHL. The included studies were: JO29138 (JAPICCTI‐142580; Japanese phase 1 study of pola monotherapy) [19]; DCS4968g (NCT01290549; global phase 1 study of pola alone or in combination with rituximab) [8]; GO27834 (NCT01691898; phase 1b/2 study of pola with rituximab or obinutuzumab) [20–22]; GO29044 (NCT01992653; phase 1b/2 study of pola with R-CHP or G-CHP [obinutuzumab, cyclophosphamide, doxorubicin, and prednisone]) [23]; and GO29365 (NCT02257567; phase 1b/2 study of pola-BR or pola with bendamustine plus obinutuzumab) [9] (Supplementary Table 1 in Online Resource 1). In these studies, pola was administered as a single agent or in combination at various dose levels by IV infusion over 30 − 90 min; full details of the methods used in these trials were previously reported [8, 9, 19–23] and study dosing is provided in Supplementary Table 1 in Online Resource 1. The studies were conducted in accordance with the Declaration of Helsinki, Good Clinical Practice guidelines, and all applicable local laws and regulations. The study protocols and their amendments, and other study-related materials, were approved by institutional review boards/ethics committees at participating centers. Written informed consent was provided by all patients for each study.

**Fig. 1:**
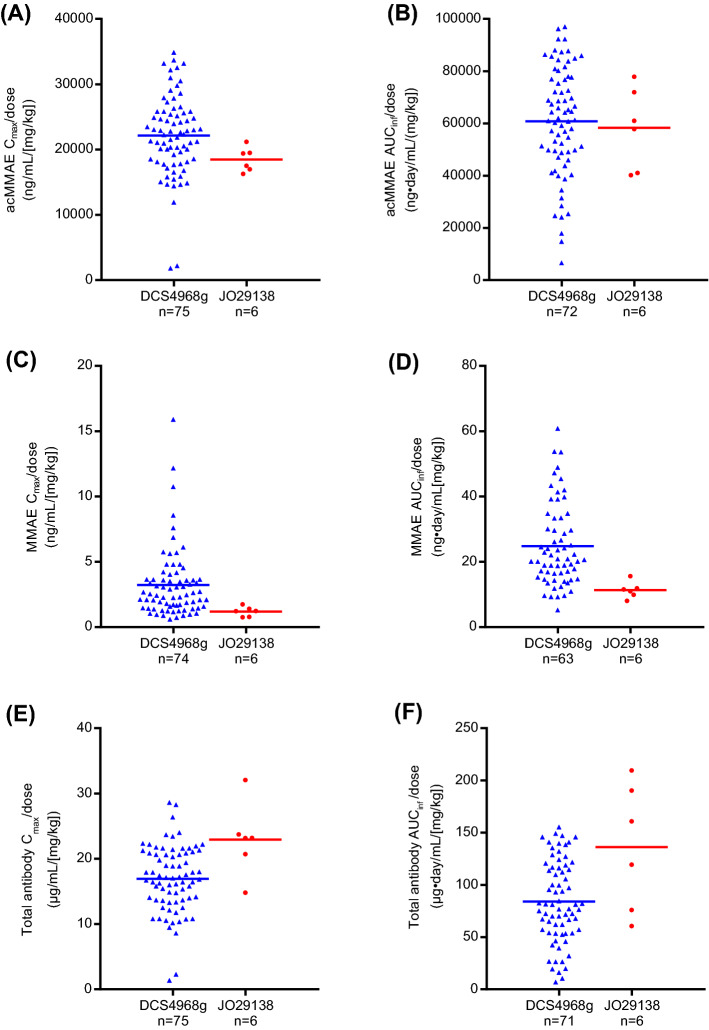
**a** Dose-normalized *C*_max_ for acMMAE; (**b**) dose-normalized AUC_inf_ for acMMAE; (**c**) dose-normalized *C*_max_ for MMAE; (**d**) dose-normalized AUC_inf_ for MMAE; (**e**) Dose-normalized *C*_max_ for total antibody; (**f**) dose-normalized AUC_inf_ for total antibody in Cycle 1 in DCS4968g (NCT01290549) and JO29138 (JAPICCTI‐142580) studies following pola monotherapy, by NCA. Blue triangles represent individual patients from study DCS4968g (NCT01290549; 0.1–2.4 mg/kg), and red circles represent individual patients from study JO29138 (JAPICCTI‐142580; 1.0–1.8 mg/kg). Horizontal lines represent the mean values. *acMMAE* antibody-conjugated monomethyl auristatin E, *AUC*_inf_ area under the concentration–time curve from time 0 to infinity, *C*_max_ maximum concentration observed, *MMAE* monomethyl auristatin E, *pola* polatuzumab vedotin

The described pola study populations supported three analyses according to the following blueprint:

1. Following pola monotherapy, exposure from PK-evaluable patients from JO29138 (JAPICCTI‐142580; (Japanese phase 1 study, *n* = 6) and DCS4968g (NCT01290549; global phase 1 study, *n* = 75) were compared as a) dose-normalized exposure (results shown in Fig. 1) and b) PK parameters from doses 1.0 mg/kg and 1.8 mg/kg (results shown in Table 1), by non-compartmental analysis (NCA). In study DCS4968g (NCT01290549), the pola monotherapy dose-escalation/expansion portion included a range of doses from 0.1 to 2.4 mg/kg with only *n* = 3 in 1.0 mg/kg and *n* = 6 in 1.8 mg/kg in B-NHL patients. Therefore, the patient numbers from DCS4968g (NCT01290549) in Fig. 1 and Table 1 are different, given that Fig. 1 includes dose-normalized PK data from all dose levels (0.1, 0.25, 0.5, 1.0, 1.8, 2.4 mg/kg).

**Table 1:** Summary of Cycle 1 PK parameters of acMMAE, total antibody, and unconjugated MMAE following pola monotherapy in Japanese and global patients from studies JO29138 (JAPICCTI‐142580) and DCS4968g (NCT01290549), respectively, by non-compartmental analysis

Pola dose (mg/kg)	Study no	Patients, *n*	PK parameter: mean (standard deviation)						
acMMAE			*C*_max_, ng/mL	diff, %	AUC_inf_, ng·day/mL	diff, %	*t*_1/2_, days	*V*_ss_, mL/kg	CL, mL/day/kg
1.0	JO29138	3	315 (28.7)	− 26.1	823 (177)	− 21.6	4.43 (0.979)^a^	64.3 (21.6)	22.2 (4.24)
	DCS4968g	3	426 (78.3)		1050 (237)		4.59 (2.28)	71.1 (24.1)	17.6 (4.44)
1.8	JO29138	3	613 (67.2)	− 23.7	2250 (274)	+ 21.0	7.98 (1.21)	91.7 (9.98)	14.4 (1.84)
	DCS4968g	6	803 (233)		1860 (966)		5.05 (1.60)	89.0 (39.2)	38.2 (55.9)

Total antibody			*C*_max_, μg/mL		AUC_inf_, μg·day/mL		t_1/2_, days	V_ss_, mL/kg	CL, mL/day/kg
1.0	JO29138	3	19.6 (4.31)	− 3.0	85.3 (30.5)	+ 19.5	5.77 (2.13)^a^	61.9 (18.3)	12.7 (4.08)
	DCS4968g	3	20.2 (3.02)		71.4 (19.8)		6.74 (4.78)	110 (49.9)	14.7 (3.84)
1.8	JO29138	3	47.4 (8.96)	+ 44.5	336 (44.2)	+ 145.3	10.8 (1.01)	70.5 (7.34)	5.41 (0.747)
	DCS4968g	6	32.8 (9.92)		137 (70.7)		6.59 (2.08)	126 (63.8)	33.5 (53.6)

Unconjugated MMAE			*C*_max_, ng/mL		AUC_inf_, ng·day/mL		t_1/2_, days	t_max_, days	
1.0	JO29138	3	1.46 (0.260)	− 43.8	12.8 (2.47)	− 49.8	3.68 (0.355)^a^	3.28 (0.49)	
	DCS4968g	3	2.60 (0.747)		25.5 (12.0)		3.28 (1.09)	2.61 (1.67)	
1.8	JO29138	3	1.67 (0.471)	− 75.5	17.7 (3.18)	− 66.2	4.65 (0.762)	4.3 (1.45)	
	DCS4968g	6	6.82 (4.73)		52.3 (18.0)		3.95 (1.05)	2.49 (1.24)	

*acMMAE* antibody-conjugated monomethyl auristatin E, *AUC*_*inf*_ area under the concentration–time curve from time 0 to infinity, *CL* clearance, *C*_max_ maximum concentration observed, *diff, %* percentage difference calculated based as (JO29138 data – DCS4968g data)/DCS4968g data * 100%, *MMAE* monomethyl auristatin E, *PK* pharmacokinetics, *pola* polatuzumab vedotin, *t*_1/2_ terminal half-life, *t*_max_ time to maximum concentration, *V*_*ss*_ volume of distribution at steady state

CL and *V*_ss_ are not estimated for unconjugated MMAE because the fraction of conversion of pola to unconjugated MMAE is unknown

^a^Three PK-evaluable patients per dose level were included from study JO29138 (JAPICCTI‐142580) analysis, except for the *t*_1/2_ analysis (*n* = 4) at the 1.0 mg/kg dose

2. Pola exposure when combined with BR was analyzed and compared between Asian and non-Asian R/R DLBCL patients in the phase 1b/2 study GO29365 (NCT02257567). All of the Asian patients enrolled in GO29365 (NCT02257567) were from South Korea; these participants represented 12.5% (10/80) of the R/R DLBCL cohort in this study, by NCA.

3. A two-analyte popPK model was previously developed based on data from 460 patients with B-NHL who participated in the DCS4968g (NCT01290549), GO29365 (NCT02257567), GO27834 (NCT01691898), and GO29044 (NCT01992653) studies [24]. Analyses of Asian region and race were conducted using this popPK model.

## Data analyses

### Bioanalytical methods

As previously reported [24], the PK profile of pola was characterized by three analytes (antibody-conjugated MMAE [acMMAE], total antibody, and unconjugated MMAE). Each analyte was measured using a validated method. Immunoaffinity liquid chromatography with tandem mass spectrometry [LC–MS/MS; lower limit of quantitation (LLOQ): 0.359 ng/mL] was used to measure acMMAE. An enzyme-linked immunosorbent assay (ELISA) (LLOQ: 50 ng/mL) was used to measure total antibody, and a LC–MS/MS (LLOQ: 0.0359 ng/mL) method was used to measure unconjugated MMAE.

### PK sampling schedules

Full details of PK sampling schedules are shown in Supplementary Table 1 in Online Resource 1.

Blood samples for the PK analyses in the JO29138 (JAPICCTI‐142580; Japanese phase 1) study were collected at Cycle 1 Day 1 (pre-dosing, 30 min, 4 h after dosing) Days 2, 4/5, 8, 11, and 15; at Cycle 2 Day 1 (pre-dosing, 30 min, 4 h after dosing); at Cycles 3–8 and Cycle 12 Day 1 (pre-dosing, 30 min after dosing) and on Days 8 and 15 of Cycles 2–4, and Day 15 of Cycle 8. Final sampling occurred 28 days after the last pola dose.

For the DCS4968g (NCT01290549; global phase 1 study of pola) study, blood samples for pola monotherapy PK were collected at Cycle 1 Day 1 (pre-dosing, 30 min, 4 h, 24 h), Day 4, Day 8, Day 11 and Day 15; at Cycle 2, Day 1 (pre-dosing, 30 min, 4 h), Day 8 and Day 15; at Cycles 3–4 Day 1 (pre-dosing, 30 min), Day 8 and Day15; Cycles 5–8 on Day 1 (pre-dosing, 30 min) and Day 15. Samples were also collected at Cycle 12 (Day 15), and every fourth cycle thereafter.

For the GO29365 (NCT02257567; global phase 1b/2 study of pola) study, blood samples for PK at the safety run-in were collected at Cycle 1, Day 2 (pre-dosing, 30 min), Day 8 and Day 15; Cycle 2 Day 1; Cycle 4 Day 1 (pre-dosing and 30 min). For pola PK at phase 2 randomization/expansion stages, samples were collected at Cycle 1 Day 2 (pre-dosing, 30 min); Cycle 2 Day 1 pre-dosing, Cycle 4 Day 1 (pre-dosing and 30 min) to treatment completion.

### PK analysis methods

NCA analysis was performed for each analyte of total antibody, acMMAE and unconjugated MMAE, as described below. NCA parameters were reported for all three analytes using Phoenix WinNonlin version 6.4 (Pharsight, Inc., Mountain View, CA). Due to the high correlation between total antibody and acMMAE, popPK analyses focused mainly on acMMAE and unconjugated MMAE, using the acMMAE-unconjugated MMAE integrated population PK model [24]. These analyses were used to assess PK differences in patients from Asia versus non-Asia regions, as well as in Asian versus non-Asian patients. acMMAE was considered the key analyte of interest for correlating with safety/efficacy, but unconjugated MMAE may also correlate with safety.

Three PK analyses were conducted for the ethnicity sensitivity assessment. First, the observed Cycle 1 dose-normalized maximum concentration (*C*_max_) and AUC from time 0 to infinity (AUC_inf_) of the three analytes in the Japanese phase 1 study JAPICCTI‐142580 and the global phase 1 study DCS4968g (NCT01290549) following pola monotherapy were compared by NCA approach. These two studies were chosen for the comparison since they are the only studies of pola monotherapy and similar PK sampling schemes were used. Of note, only one Asian patient was enrolled in the global study DCS4968g (NCT01290549), and this patient was recruited from a non-Asia clinical site. Second, we compared the observed *C*_max_ and trough concentration (*C*_trough_) on Cycle 1 and Cycle 4 of the three analytes by region (Asia, non-Asia, all), following administration of pola 1.8 mg/kg + BR Q3W in patients enrolled in the GO29365 (NCT02257567) study. Observed PK in GO29365 (NCT02257567) were compared by region only (Asia, non-Asia, all), rather than by race. Lastly, the two-analyte popPK model [24] was used to assess patients’ exposure to acMMAE and unconjugated MMAE by region (Asia, non-Asia, all) and race (Asian, non-Asian, all) using data from DCS4968g (NCT01290549), GO29365 (NCT02257567), GO27834 (NCT01691898), and GO29044 (NCT01992653) (Supplementary Table 1 in Online Resource 1). JO29138 (JAPICCTI‐142580) was not included in the popPK analysis because the study was ongoing at the time.

The popPK analysis was conducted via nonlinear mixed-effects modeling with Nonlinear Mixed-Effect Modeling (NONMEM) software, version 7.3.0 (ICON Development Solutions, Ellicott City, MD). Simulation of PK exposures based on individual empirical Bayes estimates of parameters was conducted. The partial covariate-corrected (pCC) exposure for acMMAE and unconjugated MMAE (AUC and *C*_max_ at Cycle 6 [IV pola 1.8 mg/kg Q3W]) was simulated for the comparison. The Cycle 6 exposures were theoretically the maximum exposures for the proposed dosing regimen of up to six cycles and were selected for comparison since this is likely to be less influenced by B-cell counts and disease status than the Cycle 1 exposure; therefore, comparing Cycle 6 exposure would more accurately reflect true differences among different races/regions. The pCC method used individual values of random effects and individual values of covariates, except that all patients were assumed to have R/R disease and receive pola in combination with rituximab or obinutuzumab. For each exposure metric, the geometric means ratio (GMR) and 90% confidence interval of the GMR were derived using the data from non-Asian patients or patients from non-Asia regions as reference. Visual predictive check plots were created according to geographic region and race to allow comparison between the model predicted concentrations and the observed PK data from patients with Asian race or from Asia regions. Further information on the popPK model was published previously [24].

## Results

### Demographics

Patient demographics and baseline characteristics for each study are reported elsewhere [8, 9, 19–23]; they were generally similar across treatment arms and ethnic subgroups. The numbers of Asian and non-Asian patients by race and region for the five studies are shown in Supplementary Table 1 (Online Resource 1). For NCA, the PK-evaluable population was comprised of six Japanese patients from JO29138 (JAPICCTI‐142580) and 75 global patients who were PK evaluable from DCS4968g (NCT01290549). For comparison of PK between patients enrolled in Asia and non-Asia sites in the GO29365 (NCT02257567) trial, data from 10 and 124 patients, respectively, were analyzed. A total of 18 Asian and 442 non-Asian patients (and 10/450 patients from Asia/non-Asia sites; excluding study JAPICCTI-142580) were included in the popPK-based ethnicity sensitivity assessment by race and region. The majority of Asian patients in the popPK analysis (83%) were from the GO29365 (NCT02257567) trial.

### Comparison of pola PK parameters in Japanese and global patients by NCA

This analysis compared the PK parameters in evaluable patients with B-NHL from the Japanese JO29138 (JAPICCTI‐142580; *n* = 6) study and the global DCS4968g (NCT01290549; *n* = 75) study. The Cycle 1 dose-normalized *C*_max_ and AUC_inf_ (0.1–2.4 mg/kg for DCS4968g [NCT01290549]; 1.0 and 1.8 mg/kg for JO29138 [JAPICCTI‐142580]) were compared. As shown in Fig. 1, the dose-normalized *C*_max_ and AUC_inf_ of acMMAE, the key analyte driving exposure-safety/efficacy relationships, were largely comparable (< 20% differences) between the two study populations for acMMAE (17% and 4% lower for dose-normalized *C*_max_ and AUC_inf_, respectively, based on the mean values; Fig. 1a, d); dose-normalized unconjugated MMAE *C*_max_ and AUC_inf_ appears slightly lower in JO29138 (JAPICCTI‐142580); 63% and 54% lower for dose-normalized *C*_max_ and AUC_inf_, respectively, based on the mean value (Fig. 1b, e); dose-normalized total antibody *C*_max_ and AUC_inf_ was slightly higher in JO29138 (JAPICCTI‐142580); 35% and 62% higher for dose-normalized *C*_max_ and AUC_inf_, respectively, based on the mean value (Fig. 1c, f).

Given 1.0 and 1.8 mg/kg were tested in both studies, PK parameters by NCA for the three analytes at Cycle 1 were summarized and compared for the two-dose levels (Table 1). Cycle 1 PK parameters were used for this analysis because the intensity of PK sample collection during this cycle was similar in both studies. For the three analytes, the PK comparisons between the two studies based on 1.0 and 1.8 mg/kg dose levels reached similar conclusions to the dose-normalized analysis. Comparing the JO29138 (JAPICCTI‐142580) study with the DCS4968g (NCT01290549) study, for *C*_max_ there was a 26% and 24% decrease in acMMAE; a 3% decrease and 45% increase in total antibody; and a 44% and 76% decrease in unconjugated MMAE in the patients receiving pola 1.0 and 1.8 mg/kg, respectively. Using the same comparison for AUC_inf,_ there was a 22% and 21% increase in acMMAE; a 20% and 145% increase in total antibody; and a 50% and 66% decrease in unconjugated MMAE in the patients receiving pola1.0 and 1.8 mg/kg, respectively (Table 1).

These observed numerical differences exposures from dose-normalized analysis and analysis by dose levels are potentially due to the limited number of patients in the JO29138 (JAPICCTI‐142580) study. A phase II study of 35 Japanese patients (JO40762) further confirmed the overall similar PK of pola-related analytes between the Japanese and the global patient population, which will be reported separately (publication in preparation).

### Comparison of PK in patients enrolled in Asia (South Korea) and non-Asia sites, and in all patients enrolled in GO29365 (NCT02257567) by NCA

In this analysis, with the goal of understanding the impact of ethnicity on PK after single and multiple doses of pola, both Cycle 1 and Cycle 4 PK parameters were compared for patients enrolled in and outside of Asia; Cycle 4 was the last cycle with PK sample collection at *C*_max_ during the six cycles of pola-BR treatment. The PK after multiple doses is also believed to have less inter-patient variability than Cycle 1 due to linear time-dependent exponentially declining clearance, which was starting to be negligible from Cycle 2 onwards [24].

**Fig. 2:**
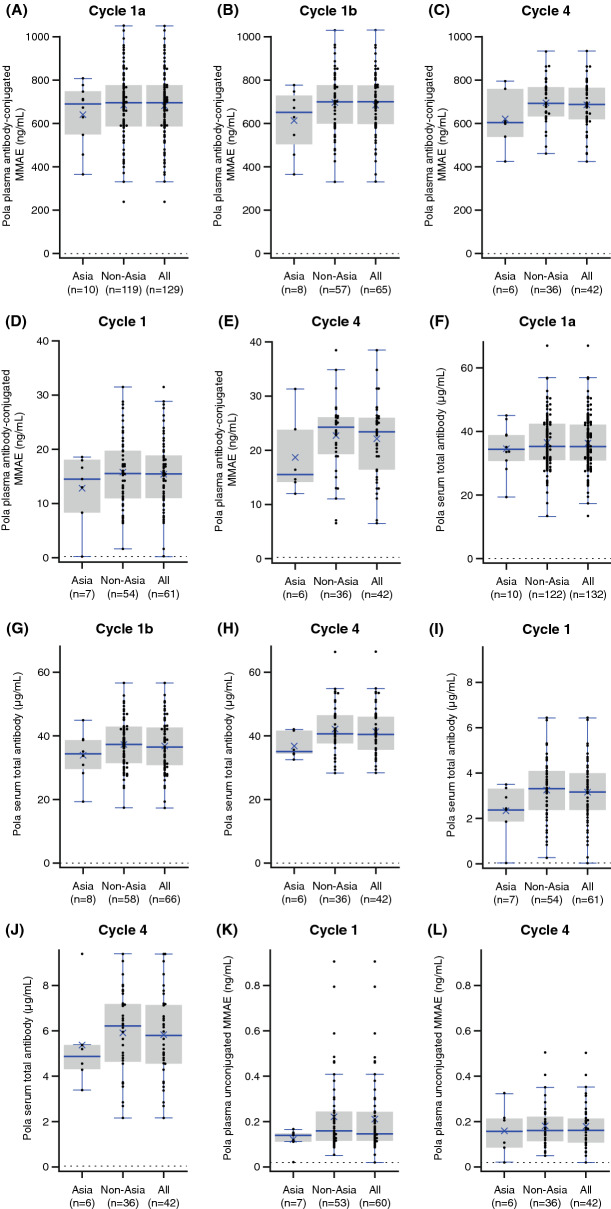
*C*_max_ of acMMAE for (**a**) Cycle 1a^a^, (**b**) Cycle 1b^a^, (**c**) Cycle 4; *C*_trough_ of acMMAE for (**d**) Cycle 1, (**e**) Cycle 4; *C*_max_ of total antibody for (**f**) Cycle 1a, (**g**) Cycle 1b, (**h**) Cycle 4; *C*_trough_ of total antibody for (**i**) Cycle 1, (**j**) Cycle 4; and *C*_trough_ of unconjugated MMAE in (**k**) Cycle 1, (**l**) Cycle 4, by region (GO29365; NCT02257567), based on NCA. R/R DLBCL and R/R FL: **a** and **f**; R/R DLBCL only: **b**–**e**, **g**–**l**. Cycle 1 trough is defined as the Day 21 sample, and Cycle 4 trough is defined as Cycle 4 pre-dose sample. Box: first and third quartiles; middle bar: median; black star: mean; black circle: observed value; dashed line: LTR (LTR values were replaced with 1/2 LLOQ values [0.180 ng/mL]); whiskers: largest observed value within the upper fence (third quartile plus 1.5 × IQR); smallest observed value within the lowest fence (first quartile plus 1.5 × IQR). Number of subjects that had ≥ 1 parameter for this analysis are Asia, *n* = 10; non-Asia, *n* = 121; and all, *n* = 131. ^a^Cycle 1a results included all patients with R/R DLBCL or FL, whereas Cycle 1b results included only R/R DLBCL patients. *acMMAE* antibody-conjugated monomethyl auristatin E, *C*_max_ maximum concentration observed, *C*_trough_ trough concentration, *DLBCL* diffuse large B-cell lymphoma, *FL* follicular lymphoma, *IQR* interquartile range, *LLOQ* lower limit of quantitation, *LTR* lower than reportable, *MMAE* monomethyl auristatin E, *pola* polatuzumab vedotin, *R/R* relapsed/refractory

The observed mean exposure (*C*_max_ and/or *C*_trough_) of acMMAE and total antibody, and the mean *C*_trough_ of unconjugated MMAE, at Cycle 4 were numerically lower (~ 9.0 to 17.5%) in patients enrolled in Asia (*n* = 10) than in those enrolled outside of Asia (*n* = 124; Table 2, Fig. 2); however, the differences were within inter-subject variability (11–38%), with overlapping ranges of exposure for patients from Asia and non-Asia regions (Fig. 2).

**Table 2:** Summary of Cycle 1 and Cycle 4 PK parameters of acMMAE, total antibody, and unconjugated MMAE by region following administration of pola 1.8 mg/kg in study GO29365 (NCT02257567), by non-compartmental analysis

Cycle no	PK parameter^a^	Region of enrollment		
acMMAE		Non-Asia (*n* = 124)	Asia (*n* = 10)	All (*n* = 134)
1	*C*_max_, ng/mL	685 (155)	643 (144)	682 (154)
	*n*	119	10	129
4	*C*_max_, ng/mL	698 (111)	622 (138)	687 (116)
	*n*	36	6	42
1	*C*_trough_, ng/mL	15.6 (6.59)	12.7 (6.53)	15.3 (6.60)
	*n*	54	7	61
4	*C*_trough_, ng/mL	22.8 (7.09)	18.8 (7.44)	22.2 (7.18)
	*n*	36	6	42

Total antibody		Non-Asia (*n* = 124)	Asia (*n* = 10)	All (*n* = 134)
1	*C*_max_, μg/mL	36.4 (8.73)	34.6 (7.60)	36.2 (8.64)
	*n*	122	10	132
4	*C*_max_, μg/mL	42.0 (8.28)	36.7 (4.06)	41.2 (8.01)
	*n*	36	6	42
1	*C*_trough_, μg/mL	3.25 (1.34)	2.34 (1.17)	3.14 (1.34)
	*n*	54	7	61
4	*C*_trough_, μg/mL	5.90 (1.85)	5.37 (2.10)	5.83 (1.87)
	*n*	36	6	42

Unconjugated MMAE		Non-Asia (*n* = 55)	Asia (*n* = 7)	All (*n* = 62)
1	*C*_trough_, ng/mL	0.219 (0.174)	0.119 (0.0488)	0.208 (0.167)
	*n*	53	7	60
4	*C*_trough_, ng/mL	0.180 (0.0985)	0.158 (0.110)	0.177 (0.0990)
	*n*	36	6	42

*acMMAE* antibody-conjugated monomethyl auristatin E, *C*_*max*_ maximum concentration observed, *C*_*trough*_ trough concentration, *MMAE* monomethyl auristatin E, *n* number of patients with available data for a given parameter at this time point, *N* number of patients that had at least one PK polatuzumab vedotin analyte sample, *PK* pharmacokinetic, *pola* polatuzumab vedotin, *SD* standard deviation

^a^Data represent mean (SD) of PK-evaluable patients

### Comparisons based on popPK simulations

The exposure (AUC, *C*_max_) of acMMAE and unconjugated MMAE at Cycle 6 following pola 1.8 mg/kg Q3W dosing was simulated by the popPK approach to assess the ethnicity effect for patients of Asian race or from Asia regions. In the popPK model, race (Asian vs. non-Asian) was identified as a statistically significant covariate for central volume (*V*_1_) of acMMAE, with 7.1% lower *V*_1_ in Asian patients than in non-Asian patients [24]. *C*_max_ was similar in Asian (*n* = 18) and non-Asian (*n* = 442) patients. Based on the pCC simulation, the difference in the simulated Cycle 6 *C*_max_ and AUC for acMMAE in Asian (*n* = 18) versus non-Asian (*n* = 442) patients was similar based on empirical Bayes estimates. Exposure to unconjugated MMAE was slightly lower in Asian patients than in non-Asian patients (16.9% lower for *C*_max_, 18.6% lower for AUC_inf_). However, the magnitude of the difference was smaller than the coefficient of variation (CV%) of unconjugated MMAE exposure, which was approximately 38–49% (Table 3).

**Table 3:** Comparison of pCC Cycle 6 exposures by race and region based on the popPK model

PK parameter	Statistic	Race		Region	
		Non-Asian (*N* = 442)	Asian (*N* = 18)	Non-Asia (*N* = 450)	Asia (*N* = 10)
acMMAE					
AUC_inf_, ng·day/mL	GM (%CV)	2910 (21%)	2870 (12%)	2910 (21%)	2800 (12%)
	GMR (90% CI)		0.988 (0.94–1.04)		0.961 (0.897–1.03)
*C*_max_, ng/mL	GM (%CV)	734 (15%)	733 (13%)	735 (15%)	691 (13%)
	GMR (90% CI)		0.998 (0.944–1.05)		0.939 (0.87–1.01)
Unconjugated MMAE					
AUC_inf_, ng·day/mL	GM (%CV)	21.4 (49%)	17.4 (40%)	21.3 (50%)	16 (25%)
	GMR (90% CI)		0.814 (0.688–0.963)		0.75 (0.647–0.87)
*C*_max_, ng/mL	GM (%CV)	1.98 (45%)	1.64 (38%)	1.98 (45%)	1.52 (23%)
	GMR (90% CI)		0.831 (0.708–0.975)		0.768 (0.669–0.881)

*acMMAE* antibody-conjugated monomethyl auristatin E, *AUC*_*inf*_ area under the concentration–time curve from time 0 to infinity, *CI* confidence interval, *C*_*max*_ maximum concentration observed, *CV* coefficient of variation, *GM* geometric mean, *GMR* geometric mean ratio, *MMAE* monomethyl auristatin E, *pCC* partial covariate-corrected, *PK* pharmacokinetic, *popPK* population PK

Similarly, the pCC simulation indicated that, by region, acMMAE exposures (*C*_max_ and AUC_inf_) in patients from Asia regions (*n* = 10) were comparable with those in patients from non-Asia regions (*n* = 450). Unconjugated MMAE exposures were lower in patients from Asia versus non-Asia regions; *C*_max_ was 23.3% lower and AUC_inf_ was 25% lower in patients from Asia regions compared with non-Asia regions (Table 3). The magnitude of these differences by race was small compared with the CV% of unconjugated MMAE exposure, which is approximately 23–50% (Table 3). Furthermore, as shown in Fig. 1, dose-normalized *C*_max_ and AUC_inf_ (0.1–2.4 mg/kg for DCS4968g [NCT01290549]; 1.0 and 1.8 mg/kg for JO29138 [JAPICCTI‐142580]) were largely overlapping despite some numerical differences.

**Fig. 3:**
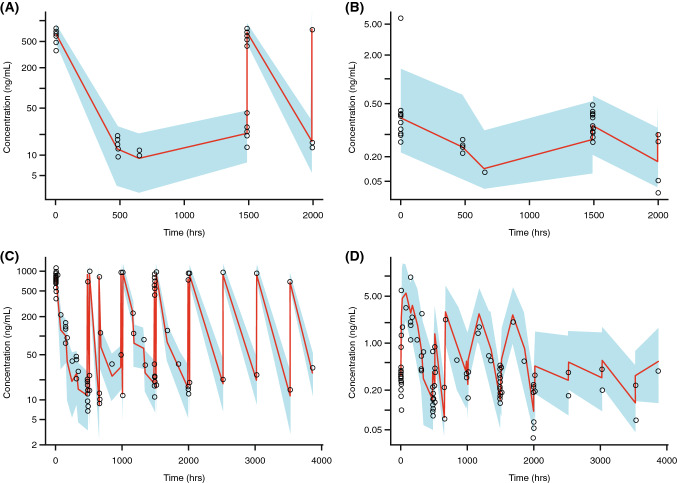
PopPK analysis: prediction-corrected visual predictive check for (**a**) acMMAE and (**b**) unconjugated MMAE in 10 patients from Asia regions (study GO29365 [NCT02257567]), and for (**c**) acMMAE and (**d**) unconjugated MMAE in 18 patients of Asian ethnicity (studies DCS4968g [NCT01290549], GO29365 [NCT02257567], GO27834 [NCT01691898], and GO29044 [NCT01992653]). Figures represent prediction-corrected observed concentrations in Asian patients (region and race) plotted versus time by popPK model based on non-Asian patients. Red line: predicted median; blue shading: predicted 80% confidence intervals; black circle: observed value. *acMMAE* antibody-conjugated monomethyl auristatin E, *MMAE* monomethyl auristatin E, *popPK* population pharmacokinetics

Prediction-corrected visual predictive check plots (Fig. 3) showed that observed concentration–time profiles for patients enrolled in Asia (*n* = 10; South Korea only from GO29365 [NCT02257567]) and Asian patients (*n* = 18; from multiple studies) were largely within the prediction interval generated from the popPK simulation. These data suggest that the popPK model could adequately predict the central tendency and variability of acMMAE and unconjugated MMAE concentrations across regions and race.

## Discussion

In this study, we conducted an ethnic sensitivity assessment of pola PK to evaluate whether the clinical recommended dose of pola in global populations is appropriate in Asian populations with B-NHL. Overall, our results indicate that there was minimal difference in pola exposure between Asian and non-Asian patients. The comparable acMMAE exposure between Asian (South Korean) patients and global patients in the phase 1b/2 GO29365 (NCT02257567) study, between Japanese patients and global patients in the phase 1 studies (JO29138 [JAPICCTI‐142580] and DCS4968g [NCT01290549]), as well as simulation evaluation by popPK model (including four studies) by race and region, suggest that a pola dose of 1.8 mg/kg Q3W would be expected to have similar PK (and, therefore, efficacy/safety) in Asian and non-Asian patients.

Exposure-efficacy analyses of pola [25] indicate that race or region are not significant covariates of this relationship, suggesting that exposure–response is likely to be similar in Asian and global patients. There were no statistically significant correlations between unconjugated MMAE exposure and multiple efficacy endpoints. However, there were significant correlations between acMMAE and efficacy endpoints, which indicated that acMMAE is the key driver for efficacy among the three analytes of acMMAE, total antibody, and unconjugated MMAE.

Similarly, exposure–response analyses of safety [25] suggested that the incidence of grade ≥ 2 peripheral neuropathy increased significantly with increasing acMMAE exposure and the incidence of grade ≥ 3 anemia increased significantly with increasing unconjugated MMAE exposure. Given that acMMAE exposure was comparable in the three PK analyses conducted in this manuscript, grade ≥ 2 peripheral neuropathy risk is expected to be similar in Asian and global patients.

For unconjugated MMAE, the current analyses identified numeric differences in the lower *C*_max_ and AUC_inf_ in Japanese patients compared with global patients. The prescribing information for pola states that “in mild hepatic impairment (aspartate aminotransferase or alanine aminotransferase > 1.0 to 2.5 × upper limit of normal [ULN] or total bilirubin > 1.0 to 1.5 × ULN), there was a 40% increase in unconjugated MMAE exposure, which was not deemed clinically significant” [26]. Based on the exposure-safety relationship for grade ≥ 3 anemia, although higher unconjugated MMAE exposure was correlated with a higher risk of this event, at a typical clinical exposure level a 40% AUC increase is associated with < 5% increase in incidence. Similarly, a 60% decrease in MMAE exposure is associated with < 5% decrease in grade ≥ 3 anemia. Consequently, the magnitude of the difference observed for unconjugated MMAE in Asian patients is unlikely to be clinically relevant to the safety of pola in Asian patients.

CYP3A4 polymorphism is not expected to impact the exposure of unconjugated MMAE in such a way that would affect the safety of pola. The activity of CYP3A4 can vary by a factor of 10 and 100 between individuals [27]. Causes of variations in CYP3A4 activity are largely non-genetic, according to the Dutch Pharmacogenetics Working Group guideline on polymorphism of CYP3A4 [27]. There is a low prevalence of CYP3A4 poor metabolizer genotypes (< 1.1%), with an overall lack of clinical evidence for quantitative association with increased plasma exposure [26]. This is further supported by the absence of CYP3A4 in the Clinical Pharmacogenomics Implementation Consortium guidelines [28]. Furthermore, given that unconjugated MMAE is both metabolized and excreted unchanged into bile [Genentech Inc., data on file], the impact of poor metabolizer genotypes on exposure is expected to be lower for pola than for drugs that are exclusively eliminated via CYP3A metabolism. Overall, the risk for a marked increase in MMAE exposure in poor metabolizers is considered low, based on the absence of clear data indicating a clinically relevant effect and the alternative non-CYP mediated pathway for elimination.

Only a small magnitude of difference was observed in the PK parameters by race and region in the popPK model (using data from a total of 460 patients with B-NHL), which were not considered relevant to pola treatment outcomes. The popPK model identified bodyweight as the most important covariate influencing PK parameters, providing support for body weight-based dosing [24]. Bodyweight correlated with race and region. Both acMMAE and unconjugated MMAE exposures were influenced by body weight to a similar extent. For acMMAE, among all the statistically significant covariates, body weight was the most important covariate which explained 41.6% and 52.0% of the inter-individual variance (IIV) for CL_INF_ and *V*_1_, compared with the base model. For unconjugated MMAE, among the statistically significant covariates, body weight was the most important covariate and explained 35.7% and 44.1% of the IIV for CL_INF_ and *V*_1_, compared with the base model. All covariates together explained the majority of the IIV (54.8% and 70.3% of CL_INF_ and *V*_1_) [24]. The pCC-based simulation is based on individual patient body weights, thus has incorporated the potential impact of lower body weight in Asian patients or Asia regions on PK exposures compared with other groups. Therefore, the pCC approach provided a realistic assessment of exposures in Asian patients or patients from Asia, in comparison with the other groups.

Previously published studies have found no impact of Asian ethnicity on clinical outcomes in DLBCL patients receiving pola or standard chemoimmunotherapy. For example, Hatake and colleagues [19] reported no substantial differences in antitumor response to pola monotherapy in Asian versus global or non-Asian populations when data from the Japanese JO29138 (JAPICCTI‐142580) and global DCS4968g (NCT01290549) studies were analyzed. Another study comparing PFS and OS in global and Asian patients with DLBCL following treatment with R-CHOP reported no marked differences in treatment outcomes [14]. As far as we are aware, there are no reports of clinically meaningful differences in treatment outcomes in Asian versus global patients treated with CHOP. Interestingly, Asian ethnicity has also been reported to have no effect on the PK of another ADC, trastuzumab emtansine, in patients with HER2-positive breast cancer, suggesting that treatment outcomes in these patients would be similar to those of non-Asian populations [29].

## Conclusions

This study shows that PK data for Asian (South Korean) patients were similar to those in non-Asian patients in a phase 1b/2 pola-BR cohort of patients with R/R DLBCL, with the overlapping distribution of exposure from a Japanese phase 1 study and global phase 1 study of pola monotherapy in B-NHL. In addition, the popPK model analyses found no clinically meaningful differences in acMMAE and unconjugated MMAE exposures after pola dosing in Asian versus global populations with B-NHL, when analyzed by both race and region. The small magnitude of difference in PK observed is not considered to be clinically relevant, supported by exposure–response relationships of efficacy and safety. Overall, our data indicate that no adjustment of the clinically recommended pola dose of 1.8 mg/kg Q3W in Asian patients is warranted in the currently approved R/R DLBCL indication, or in other B-NHL indications that could potentially be approved in the future. This dosing schedule is being utilized in an ongoing phase 2 study of pola-BR in Japanese patients with R/R DLBCL (JO40762).

## Electronic supplementary material

Below is the link to the electronic supplementary material.

Supplementary file1 (DOCX 24 kb)

## Data Availability

“Qualified researchers may request access to individual patient-level data through the clinical study data request platform (https://vivli.org/). Further details on Roche's criteria for eligible studies are available here (https://vivli.org/members/ourmembers/). For further details on Roche's Global Policy on the Sharing of Clinical Information and how to request access to related clinical study documents, see here**:** (https://www.roche.com/research_and_development/who_we_are_how_we_work/clinical_trials/our_commitment_to_data_sharing.htm).”
