# Supplementary material for: Asian race and origin have no clinically meaningful effects on polatuzumab vedotin pharmacokinetics in patients with relapsed/refractory B-cell non-Hodgkin lymphoma
Source: Cancer Chemother Pharmacol. 2020 Aug 8;86(3):347–59. doi: 10.1007/s00280-020-04119-8 (PMC7478950; doi:10.1007/s00280-020-04119-8)
Supplement: Supplementary file 1 — Supplementary file1 (DOCX 24 kb) [file 280_2020_4119_MOESM1_ESM.docx]

**SUPPLEMENTARY MATERIAL**

**Asian race and origin have no clinically meaningful effects on polatuzumab vedotin pharmacokinetics in patients with relapsed/refractory B-cell non-Hodgkin lymphoma**

Rong Shi^1^ · Tong Lu^1^ · Grace Ku^1^ · Hao Ding^1^ · Tomohisa Saito^2^ · Leonid Gibiansky^3^ · Priya Agarwal^1^ · Xiaobin Li^1^ · Jin Yan Jin^1^ · Sandhya Girish^1^ · Dale Miles^1^ · Chunze Li^1^ · Dan Lu^1^

^1^ Genentech Inc., South San Francisco, CA, USA

^2^ Chugai Pharmaceutical Co., Ltd., Tokyo, Japan

^3^ QuantPharm LLC, North Potomac, MD, USA

**Supplementary Table 1** Studies utilized in the pola PK analyses

| **Study no.** | **Disease and setting** | **Pola dose/schedule** | **PK sampling** | **Race, n** | | **Geographic**  **Region, n** | |
| --- | --- | --- | --- | --- | --- | --- | --- |
|  |  |  |  | **Asian** | **Non-Asian^a^** | **Asia** | **Non-Asia^b^** |
| JO29138 (JAPICCTI‐142580)^c^ | R/R B-NHL; phase 1 study in Japan | 1.0 or 1.8mg/kg every 21 days for 8 cycles | C1D1 0, 0.5, and 4hr, C1D2, C1D4/5, C1D8, C1D11, C1D15; C2D1 0, 0.5, and 4hr; C2−4D8, C2−4D15; C3−8D1 and C12D1 0, 0.5hr; C8D15 | 6 | - | 6 | - |
| DCS4968g (NCT01290549) | R/R B-NHL, R/R CLL; global phase 1/1b study | **Phase 1 single agent**  Pola: 0.1, 0.25, 0.5, 1.0, 1.8, or 2.4 mg/kg Q3W | C1D1 0, 0.5, 4, and 24hr, C1D4, C1D8, C1D11, C1D15, C2D1 0, 0.5, and 4hr, C2D8, C2D15, C3–4D1 0 and 0.5hr, C3–4 D8, D15, C5–8D1 0 and 0.5hr, C8D15, C12, and every 4^th^ cycle beyond 0 and 0.5hr, TC/ET + PT | 1 | 76 | 0 | 77 |
|  |  | **Phase 1b combined with rituximab**  R: 375mg/m^2^  Pola: 2.4mg/kg Q3W | C1D2 0 and 0.5hr, C1D4, C1D8, C1D15, C2–4D2 0 and 0.5hr, C2–4D8, C2–4 D15, C5–8, C12, and every 4^th^ cycle after, D2 0 and 0.5hr, TC/ET + PT |  |  |  |  |
| GO27834 (NCT01691898) | R/R DLBCL, R/R FL; global phase 1b/2 study | **Phase 1b safety run in**  G: 1,000mg  Pola: 1.8mg/kg Q3W | C1D2 0 and 0.5hr, C1D8, C1D15, C2D1 0 and 0.5hr, C4D1 0 and 0.5hr, TC/ET + PT | 2 | 160 | 0 | 162 |
|  |  | **Phase 2**  R: 375mg/m2  Pola: 2.4mg/kg Q3W | C1D2 0 and 0.5hr, C1D8, C1D15, C2D2, C3D2 0 and 0.5hr, C3D8, C3D15, C4D2 0 and 0.5hr, and every 4^th^ cycle after, TC/ET + PT |  |  |  |  |
| GO29044 (NCT01992653) | First-line DLBCL; global phase 1b/2 study | **Phase 1b dose escalation**  R: 375mg/m^2^  Cyclophosphamide: 750mg/m^2^  Doxorubicin: 50mg/m^2^  Pola: 1.0, 1.4, 1.8, or 2.4mg/kg Q3W  G: 1,000mg  Cyclophosphamide: 750mg/m^2^  Doxorubicin 50mg/m^2^  Pola: 1.4 or 1.8mg/kg Q3W | C1D2 0 and 0.5hr, C1D8, C1D15, C2D2, C3D1, C4D1 0 and 0.5hr, PT | 0 | 82 | 0 | 82 |
|  |  | **Phase 2 dose expansion**  R: 375mg/m^2^  Cyclophosphamide: 750mg/m^2^  Doxorubicin: 50mg/m^2^  Pola: 1.8mg/kg Q3W  G: 1,000mg  Cyclophosphamide: 750mg/m^2^  Doxorubicin: 50mg/m^2^  Pola: 1.8 mg/kg Q3W | C1D2 0 and 0.5hr, C1D8, C1D15, C2D2, C3D1, C4D1 0 and 0.5hr, TC/ET + PT |  |  |  |  |
| GO29365 (NCT02257567) | R/R DLCBL, R/R FL; global phase 1b/2 study | **Phase 1b safety run in**  B: 90mg/m^2^  R: 375mg/m^2^  Pola: 1.8mg/kg Q3W (DLBCL) or Q4W (FL)  B: 90mg/m^2^  G: 1,000mg  Pola: 1.8mg/kg Q3W (DLBCL) or Q4W (FL) | C1D2 0 and 0.5hr, C1D8, C1D15, C2D1, C4D1 0 and 0.5hr | 15 | 124 | 10 | 129 |
|  |  | **Phase 2 randomization**  B: 90mg/m^2^  R: 375mg/m^2^  Pola: 1.8mg/kg Q3W (DLBCL) or Q4W (FL)  B: 90mg/m^2^  R: 375mg/m^2^ | C1D2 0 and 0.5 hr, C2D1 0hr, C4D1 0 and 0.5hr, TC |  |  |  |  |
|  |  | **Phase 2 expansion**  B: 90mg/m^2^  G: 1,000mg  Pola: 1.8mg/kg Q3W (DLBCL) or Q4W (FL) | C1D2 0 and 0.5hr, C2D1 0hr, C4D1 0 and 0.5hr, TC |  |  |  |  |
| **Total no. of patients in popPK analyses (race/region)** | | | | **18** | **442** | **10** | **450** |

*B* bendamustine*, B-NHL* B-cell non-Hodgkin lymphoma, *C* cycle, *CLL* chronic lymphocytic leukemia, *D* Day, *DLBCL* diffuse large B-cell lymphoma, *ET* early termination, *FL* follicular lymphoma, *G* obinutuzumab, *hr* hour, *PK* pharmacokinetic, *pola* polatuzumab vedotin, *popPK* population PK, *R* rituximab, *PT* post-treatment, *Q3W* every 21 days, *Q4W* every 28 days, *R* rituximab, *R/R* relapsed/refractory, *TC*treatment completion.

^a^Includes White and other races

^b^Includes patients enrolled from Western and Eastern Europe, North America, Pacific (non-Asia), and other regions

^c^Study not included in the popPK model analyses by race or region
